# Leaf-cutting ants’ critical and voluntary thermal limits show complex responses to size, heating rates, hydration level, and humidity

**DOI:** 10.1007/s00360-021-01413-6

**Published:** 2021-11-27

**Authors:** Cleverson Lima, André Frazão Helene, Agustín Camacho

**Affiliations:** 1 Department of Physiology, Instituto de Biociências, USP, São Paulo, SP, 05508‑090 Brazil; 2 Department of Evolutionary Ecology, Estación Biológica de Doñana, CSIC, 26 Américo Vespucio Av., 41029 Isla de la Cartuja, Spain; 3 Department of Entomology, College of Agriculture, Food, and Environment, University of Kentucky, KY 40546 Lexington, USA

**Keywords:** Voluntary thermal maximum, Body size, Dehydration, Heating rate, Relative humidity, Critical thermal maximum

## Abstract

Thermal variation has complex effects on organisms and they respond to these effects through combined behavioral and physiological mechanisms. However, it is less clear how these traits combine in response to changes in body condition (e.g., size, hydration) and environmental factors that surround the heating process (e.g., relative humidity, start temperatures, heating rates). We tested whether these body conditions and environmental factors influence sequentially measured Voluntary Thermal Maxima (VT_max_) and Critical Thermal Maxima, (CT_max_) in leaf-cutting ants (*Atta sexdens rubropilosa*, Forel, 1908). VT_max_ and CT_max_ reacted differently to changes in body size and relative humidity, but exhibited similar responses to hydration level, start temperature, and heating rate. Strikingly, the VT_max_ of average-sized workers was closer to their CT_max_ than the VT_max_ of their smaller and bigger sisters, suggesting foragers maintain normal behavior at higher temperatures than sister ants that usually perform tasks within the colony. Previous experiments based on hot plate designs might overestimate ants’ CT_max_. VT_max_ and CT_max_ may respond concomitantly or not to temperature rises, depending on body condition and environmental factors.

**Supplementary Information:**

The online version contains supplementary material available at 10.1007/s00360-021-01413-6.

## Introduction

Accurately predicting ecological responses to climate change requires a thorough understanding of how organisms perform under thermal stress in different contexts. Traditionally, this problem has been approached by comparing thermal limits under different conditions that may alter tolerance levels (e.g., Angilletta et al. 2007; Christian and Morton 1992). However, since organisms integrate behavioral and physiological thermal tolerance to deal with temperature rises (Williams et al. 2008), detailed information on behavioral responses to temperature is needed to accurately predict responses to climate warming.

The physiological performance of ectotherms in response to temperature change is described with a Gaussian thermal performance curve (Angilletta 2014; Camacho et al. 2018; Huey and Stevenson 1979—although often skewed from the standard original model, see Sinclair et al. 2016). Within voluntarily tolerated thermal levels, different aspects of physiological performance are optimized at different temperatures (e.g., stamina may be more optimized at lower temperatures than sprint speed, Huey et al. 1984). Nonetheless, if body temperatures rise excessively, locomotor and neural processes eventually stop, and the animals reach their Critical Thermal Maximum (CT_max_, Cowles and Bogert 1944; Jørgensen et al. 2020), which can kill them almost immediately (Angilletta et al. 2007; Christian and Morton 1992; Ribeiro et al. 2012).

At temperatures close to CT_max_, animals often move away from heat sources, exhibiting their Voluntary Thermal Maximum (VT_max_, Camacho et al. 2018; Cowles and Bogert 1944). This trait represents how organisms try to keep their body temperatures below too costly or dangerous levels (Martin and Huey 2008). VT_max_ may remain invariable across populations separated by millions of years (Wiens et al. 2019), and, despite being a behavioral trait, VT_max_ has been observed to change relatively little compared with CT_max_ in lizards (e.g., Camacho and Rusch 2017). Moreover, preferred temperatures correlate with CT_max_ across species (Huey and Kingsolver 1993; Sinervo et al. 2010), suggesting that thermal preference and thermal tolerance may vary being positively correlated (but not always, see Huey and Bennett 1987). In contrast, VT_max_ and CT_max_ have been found to vary independently within populations (e.g., lizards, Camacho et al. 2018). In this sense, behavioral and physiological traits of thermal tolerance, such as VT_max_ and CT_max_, might respond differently to internal and external influences, such as different body conditions and environmental factors.

Physiological thermal tolerance is influenced by both environmental conditions and inherent characteristics of the organism. Among these characteristics, body size affects many physiological (Hurlbert et al. 2008; Jensen and Nielsen 1975; Ribeiro et al. 2012) and ecological traits (Johnson 2008; Kaspari 1993). Larger body size often raises the CT_max_ of ectothermic animals (Angilletta et al. 2007; Christian and Morton 1992, Ribeiro et al. 2012, but see Oyen and Dillon 2018). Also, larger bodies tend to store more water and present lower rates of water loss (Edney 1977). In small arthropods (i.e., ants), longer limbs also increase the distance of the separation of the body from heated surfaces, which greatly reduces the heat load within millimeters (Cerdá and Retana 2000). Ant castes can be differentiated by morphological aspects (size), but individuals of overlapping size often specialize in different tasks within and out of the nest (called temporal castes, Wilson 1980). Accordingly, it could be expected that ants of different sizes may show different thermal tolerance as a result of exposure to different temperatures (Baudier and O’donnel 2018). Few studies have related thermal tolerance and body size in insects (e.g., Clémencet et al. 2010; Verble-Pearson et al. 2015; Baudier et al. 2015; Johnson and Stahlschmidt 2020) and none have integrated VT_max_ and CT_max_ before. One potential problem to explore such integration is that traditional approaches with linear models might not accurately describe the relationship of size and thermal tolerance (see Ribeiro et al. 2012 Fig. 1C, where a non-linear response of CT_max_ to size is suggested by the data). Thus, the shapes of these relationships remain unknown.

The level of body hydration (HL) can be another body condition regulating thermal tolerance among ectothermic animals. Water loss rates increase with rising body temperatures (Edney 1977; Lighton and Bartholomew 1988), and dehydration stress lowers the VT_max_ and CT_max_ of some ectotherms (e.g., Anurans, Anderson and Andrade 2017). Small arthropods also react to water stress. *Messor pergandei* ants with lower water reserves forage closer to their nests (Lighton et al. 1994), *Atta Columbica* ants select resources with higher water content (Bowers and Porter 1981), and individuals with specific smaller size from *Atta sexdens rubropilosa* are recruited to transport water to the colony when it is in hydric stress (Ribeiro and Navas 2008). Thus, dehydrated individuals might have their VT_max_ lower to protect themselves from lower CT_max_, especially if they are experiencing heat stress in drier environments (i.e., environments with lower relative humidity, RH). Yet, to our knowledge, such responses have not been evaluated in arthropods.

Other environmental traits are typical elements of the experimental setup. These are the temperature at which the animal starts to be heated (the start temperature, ST), and the rate at which temperature rises (the heating rate, HR, sometimes called ramping rates, Overgaard et al. 2012). Both may alter the risks of overheating by changing the time at which the animal is exposed to stressfully hot temperatures. They also can change the animal’s perception of risk of overheating. For example, faster heating rates could lead an organism to seek thermal refuge at lower temperatures (i.e., present lower VT_max_), in anticipation of a higher risk of exceeding their CT_max_. In contrast, slower heating rates involving longer exposures to sublethal temperatures might induce higher physiological stress (Rezende et al. 2020). If such conditions lower an organism’s CT_max_, a decrease in VT_max_ might be expected, under the premise that VT_max_ will parallel CT_max_ variation. These scenarios can be tested by manipulating heating rates (HR) and start temperatures (ST) in heat tolerance experiments (Terblanche et al. 2007; Camacho et al. 2018).

Ants play indispensable roles in mediating ecosystems' services and disservices (Del Toro et al. 2012). This makes important to understand how these animals are affected by environmental changes that may be harmful, such as climate change trends. The small size and the availability of countless individuals make ants appropriate experimental models for thermal tolerance studies. Also, the leaf-cutting ant *Atta sexdens rubropilosa* (Forel 1908), which is the focus of this study, varies in body size both within and across castes (Hölldobler and Wilson 1990), which allowed us to test hypotheses regarding body size and thermal tolerance.

Based on the topics presented above, we tested if leaf-cutting ants’ VT_max_ and CT_max_ (1) increased in parallel with body size, (2) increased or decreased similarly with both relative humidity and hydration levels, and (3) increased or decreased similarly with variations in start temperatures and heating rates.

Quantifying the effects of body conditions and environmental factors on VT_max_ and CT_max_ will help us better understand the integration of behavioral and physiological thermal tolerance. In addition, these results will inform how experimental setups influence measures of behavioral and physiological thermal tolerance.

## Materials and methods

### Study animals

The ants used in the experiments came from five colonies collected at Rio Claro, SP (Brazil), near the Laboratório de Formigas Urbanas (Center of Social Insect Studies, CEIS—Universidade Estadual Paulista). They were maintained in laboratory at 24 °C ± 1 and 55–65% relative humidity. These colonies were brought and maintained for 1–2 years at the Laboratório de Ciências da Cognição (Department of Physiology, Instituto de Biociências, Universidade de São Paulo), where the experiments were conducted. The animals were fed every day with leaves of *Acalypha *spp. These conditions have been used for over 30 years by researchers of the CEIS, a reference institution of social insect studies (more details in Bueno et al. 2002 and Ribeiro et al 2012).

### The thermal tolerance meter

We developed a device capable of sequentially measuring the VT_max_ and CT_max_ of four ants in about 15 min, the Thermal Tolerance Meter. In this device, ants are simultaneously heated in five chambers immersed in a thermal bath. This thermal bath consists of a transparent plastic box (15 × 10 × 8 cm) filled with water (1200 ml) and heated by a Magnetic Hot Plate Stirrer (Quimis Q261). These identical individual chambers are 5-ml polystyrene tubes, with 3.5 mm diameter. The chambers are horizontally inserted in the thermal bath, with the tip left outside to provide a temporal thermal refuge. The heated part of each tube has 8 cm length, while the refuge is 3 cm in length. Both openings of each tube are closed during the experiments by glass rods whose diameter fit the opening gap. Yet, they leave a very thin space which allows the thin thermocouple to pass through and record the body temperature at the model ant. Apart from closing the entrances, these rods allowed to push ants out of the thermal refuge, or the tube if necessary.

During the tests, four ants (one in each chamber) had their VT_max_ and CT_max_ measured in each run. Ants’ body temperatures were represented by that of an individual of similar size which had been killed immediately before. For that purpose, we inserted a thermocouple in this fifth individual´s thorax and placed it in the (middle) chamber after it was dead. This way, we accounted for ants’ body size and shape during the measurement of VT_max_ and CT_max_, and avoided changing its mass, as it could have happened during freezing euthanasia. For each trial, we used a different model ant, which always had a similar size to the ants being tested. The body temperatures were monitored by a T-type thermocouple (1 mm diameter, Omega ©), connected to a computer through a datalogger (Picolog® TC H8). The temperature inside the thermal refuge was monitored to assess the differences with the heated part of the chamber. The thermal dynamics and thermal heterogeneity within the heating system were assessed, and they showed satisfactorily low thermal heterogeneity (See Fig. S2 in the supplementary file https://doi.org/10.6084/m9.figshare.14414243.v2). The RH of the room ranged from 55–65%, except during the experiments which had been designed to alter hydration level and relative humidity.

### Sequential measures of ants’ VT_max_ and CT_max_ using the thermal tolerance meter

Before each experiment, the ants were acclimated to room temperature (25 °C) for 1 h (with water ad libitum). Each individual´s VT_max_ was registered as the temperature at which they entered and remained in the thermal refuge for at least 7 s. In a pilot experiment, the ants could quickly visit the thermal refuge (always less than 3–4 s). Thus, by waiting 7 s before registering the VT_max_, we ensured the ants were avoiding the heating chamber. After recording the ants’ VT_max_, the refuge was occupied by the glass rod, closing it and pushing each ant back to the heating chamber, preventing it to come back into the thermal refuge. In the heating part, ants kept moving inside the chamber until their legs became paralyzed, causing disorganized locomotion (which was visible for the observer because of the fully transparent thermal bath). The CT_max_ was then recorded and the ants were taken to a Petri dish for cooling down and observation. The ants that did not survive for at least two hours after the experiment had their VT_max_ and CT_max_ disregarded in the analyses (8% of 186 subjects tested (15) were disregarded from the analyses). At the end of each day of experiments, all tested ants were killed by decapitation and disposed.

### The dehydration treatment

First, four groups of ten ants were isolated from the colonies for a few hours in recipients with water available ad libitum. Assuming that the animals were fully hydrated, they were weighted in a semi-analytical balance (readability: 0.0001 g), which was recorded as the Initial Weight (100% of Hydration Level). Then, the ants were placed in perforated vials (1.5 ml Eppendorf tubes, with one ant inside each), which were placed within four 7 × 7 × 8 cm sealed recipients. The bottoms of two of these recipients were filled with silica gel to create a dehydrating atmosphere within the recipients. The other two recipients had water-soaked silica gel, creating a very humid atmosphere. The vials containing the ants were separated from the silica by paper towels, preventing any direct contact with ether the silica or the water. After a few hours, the final weight of each animal was recorded and its Hydration Level (HL) was obtained as the difference between the initial and final weight (in %). This procedure provided ants with a range of differently dehydrated bodies (~ 75–100%).

## Experiments

### Measuring the effects of body size on VT_max_ and CT_max_

We measured the VT_max_ and CT_max_ of 49 individuals, ranging from 1.5 to 4 mm in head width. Head width is widely used to represent leaf-cutting ants’ size (see Wilson 1980). Although some smaller individuals (0.1–1.4 mm) can be found outside the nest, most of them aid the larvae inside the fungal gardens. The 1.5–4 mm range in head width thus represents the individuals found at the foraging trails. The sizes were measured with an analogic caliper (accuracy: 0.01 mm). For this experiment, the ST was 25 °C, but the heating rate varied on an individual basis (i.e., heating rate was calculated based on how much each ant’s body temperature increased per minute during the experiment). The initial RH inside the thermal chambers during this experiment was 55–65%. Potential observer bias was evaluated in the following manner: three different observers collected the measures of the VT_max_ and CT_max_, independently, and then compared their measures. The observed differences between their independent measures remained below one degree across the 49 measures.

### Measuring the effects of hydration level and relative humidity conditions on VT_max_ and CT_max_

The VT_max_ and CT_max_ of individuals with HL ranging 75–100% were tested at ambient RH of 50% (*N* = 15) and 85% (*N* = 20), always using individuals of about 2.3 mm head width, ST = 25 °C, and HR ~ 1 °C/min. The experiments were carried out in a controlled climatized room (FITOTRON EL011—Eletrolab). The initial RH of the air and inside thermal chambers was similar to the ambient RH, as checked using a hygrometer (HT-600 Instrutherm). Measuring humidity within the thermal chambers was not possible, but even if some unnoticed variation in RH might happen during heating, it can be safely assumed that tubes at 50% remained always much drier than the ones at 85%.

### Measuring the effects of start temperature and heating rates on VT_max_ and CT_max_

We assessed these effects in two series of trials that we later pooled together for analysis. First, we measured the VT_max_ and CT_max_ of 57 individuals varying the HR between 0.5 and 3 °C/min but keeping the start temperature at 25 °C. Next, we measured 45 additional individuals, varying the ST between 23 and 32.5 °C, and heating rates between (0.6–2.6 °C/min). In this way, we ensured that ST and HR would vary independently across the full set of 102 ants, and considered both factors as continuous variables. For these trials, we used ants with about 2.3 mm in head width, taken directly from the colonies and kept for 1 h with access to water, acclimating to the room temperature (25 °C). The initial RH inside the thermal chambers was always in the range 55–65%.

### Data analyses

We fitted Linear Mixed Models (Bates et al. 2014) relating the VT_max_ or the CT_max_, separately to the described predictors (body size, heating rate, start temperature, hydration level, and relative humidity conditions). In each fitted model, either the VT_max_ or CT_max_ was the response variable, and the corresponding predictors entered as fixed factors. Ants´ colony (categorical factor with five levels) entered as grouping variable (random effect) to control the lack of independence in traits among ants of the same colony.

### Model selection

Before estimating fixed and random effects, we selected the best models describing the relationships between each response trait and its most relevant predictive factors. We used the Akaike Information Criterion (AIC, Akaike 1974), which penalizes the addition of parameters more than the Bayesian Information Criterion (Wang and Liu 2006), to choose the model that best describes such relationships. One model can be considered as having a better fit than another if its AIC value is lower by a difference of at least 2 (Wang and Liu 2006). Among models with a similar fit (difference below 2 AIC units), we chose the one with the lowest AIC, but considered the result given by the second-lowest. Given the important effect of heating rates (HR), we included this factor in all the models fitted.

To flexibly identify the relationships between body size and VT_max_ and CT_max_ in ants, we compared four models in total, ranging from first-order equations (linear) up to a fourth-order one (non-linear). As models become more complex, having a low number of categories in the fixed factor may lead to overfitting because parameters are calculated within each level of the random effects. In this case, dropping terms and changing the random effect by a fixed effect (Barr et al. 2013). Thus, we repeated this model selection procedure twice, based on simpler, generalized least squares with either colony as fixed effect, or not including colony as factor. The procedure and results can be found in the supplementary material (Table S1).

When testing for the relative importance of hydration level (HL) and relative humidity (RH) on VT_max_ and CT_max_, we used AIC to select between four competing mixed models in which colony was again the grouping factor. These models were: (1) a model containing only HL, (2) a model including HL and RH, (3) a model including both predictive variables and HR, without interactions, and (4) a model including the three terms and an interaction between HL and RH. To describe the fit of the selected model, we calculated a conditional pseudo-Rsquared value developed for mixed models (Nakagawa et al. 2017).

When testing the effect of ST and HR on VT_max_ and CT_max_, we selected four competing models. These models were: (1) a null model containing only the intercept, (2) a model including HR alone, (3) including HR and ST independently, and (4), a model including their interaction. We also added the conditional pseudo-Rsquared value for the selected model to describe this model’s fit.

All analyses were performed in the R environment (R Development Core Team 2018) using the package nlme (Pinheiro et al. 2017). Fixed Pseudo R squared was obtained using the MuMin package (Barton 2009).

## Results

### Effects of body size (BS) on VT_max_ and CT_max_

**Fig. 1:**
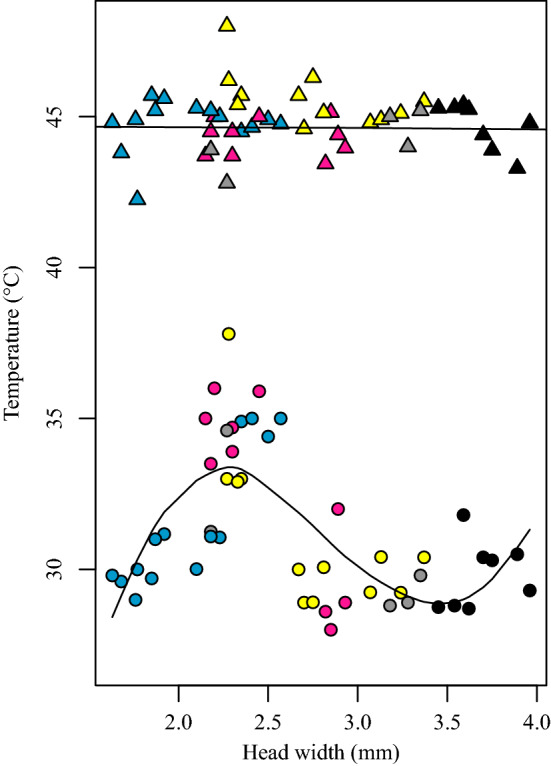
Relationships between size, VT_max_ and CT_max_. While average-sized workers present VT_max_ higher than smaller and larger workers, their CT_max_ did not increase with body size. Each point in the figure represent one ant. Circles represent VT_max_ and triangles represent CT_max_. Color/tone indicates the colony that each ant was from. The predictive lines account for heating rates in both cases (color figure online)

BS did not show linear correlations with VT_max_ (*DF* 42, *B* 0.130, SD 0.294, *t* 0.451, *p* 0.657) or CT_max_ (*DF* 42, *B* 0.185, SD 0.369, *t* 0.503, *p* 0.621), although heating rate did affect both measures (VT_max_; *DF* 42 *B* 3.466, SD 0.546, *t* 6.344, *p* 0.000; CT_max_; *DF* 42 *B* 1.014, SD: 0.368, *t* 2.755, *p* 0.008). Instead, a third-order polynomial explained the relationship between VT_max_ and size better than both simpler and more complex models (AIC difference was > 2 with second best model). The observed non-linear trend in VT_max_ was created by average-sized workers (2–2.6 mm head width) from four different colonies, whose VT_max_ reached closer to their CT_max_, compared to larger and smaller individuals of the same and other colonies (Fig. 1). This trend was also indicated by the 4th order polynom, which had the second best fit.

In turn, the CT_max_ response was not better described by more complex models compared to a straight line (Table 1). These models show that both traits respond differently to body size in leaf-cutting ants. More detailed results can be found in the supplementary material (Table S1 A for VT_max_ and B for CT_max_).

**Table 1:** Fit of different models describing the relationship of VT_max_ and CT_max_ with different factors

Predictors	*DF*	VT_max_	CT_max_
BS linear + HR	5	202.2	128.89
BS 2nd Order + HR	6	202.02	128.96
BS 3rd Order + HR	7	199.67	129.27
BS 4th Order + HR	8	201.21	131.19
RH	4	174.8	160.08
RH + HR	4	176.32	172.27
RH + HL + HR	6	159.84	144.46
RH*HL + HR	7	157.24	143.54
Intercept	3	431.64	336.53
HR	4	430.05	293.31
HR + ST	5	422.24	294.61
HR*ST	6	422.75	295.88

*BS* body size, *ST* start temperature, *HR* heating rate, *RH* relative humidity, *HL* hydration level, *DF* parameters estimated

^+^Indicates independent terms

^*^Indicates interaction between terms

### Combined effects of hydration level (HL) and relative humidity (RH) on VT_max_ and CT_max_

**Fig. 2:**
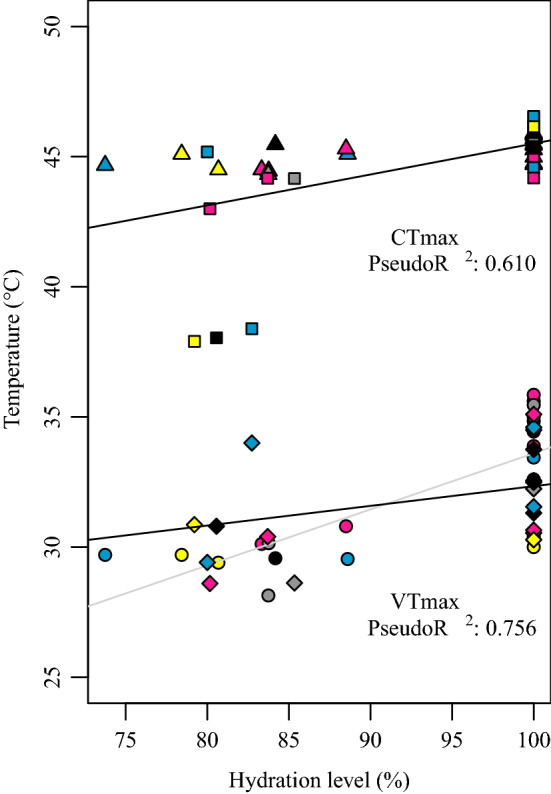
Effects of hydration state and relative humidity on CT_max_ (above) and VT_max_ (below). Ants’ VT_max_ was more reactive to hydration level when heated in a humid environment (85% relative humidity, grey line, lozenges), compared to that of ants heated in a drier environment (50% relative humidity, black line, circles). Meanwhile, ants’ CT_max_ was similarly reactive to hydration level in either a more humid (85% relative humidity, black line, squares) or drier environment (50% relative humidity, triangles)

The competing models that we compared here were: (1) HL, (2) RH plus HL (3) the three terms, independent, (4) the three terms, with the interaction between HL and RH. Regarding the VT_max_, model 4 was the most likely by a difference > 2 in AIC values with the second best (Table 1). The model detects a positive but very weak interaction between HL and RH, where individuals were more reactive to HL within the humid treatment (*N* 40, *B* 0.106, SD 0.050, *t* 2.110, *p* 0.042). In general, VT_max_ increased with HL (*N* 40, *B* 0.079, SD: 0.035, *t* 2.264, *p* 0.031) and HR (*N* 40, *B* 7.414, SD 3.118, *t* 2.377, *p* 0.023), but the effect of RH was not statistically significant (*N* 40, *B* − 8.761, SD 3.936, *t* − 194, *p* 0.061). See observations and resulting trends in Fig. 2. The full output of this model can be found in Table S2 A (Table 2).

**Table 2:** Model coefficients table

Trait	Effect	*DF*	*B*	SD	*t*	*p* value
VTmax	BS on VT_max_	42	0.13	0.294	0.451	0.657
	BS and HR on VT_max_	42	3.466	0.546	6.344	0
	HL and RH on VT_max_	40	0.106	0.05	2.11	0.042
	HL on VT_max_	40	0.079	0.035	2.264	0.031
	HL and HR on VT_max_	40	7.414	3.118	2.377	0.023
	RH on VT_max_	40	− 8.761	3.936	− 194	0.061
	ST on VT_max_	101	0.134	2.467	1.47	0.143
	HR on VT_max_	101	1.313	0.373	3.516	0.001
CTmax	BS on CT_max_	42	0.185	0.369	0.503	0.621
	BS and HR on CT_max_	42	1.014	0.368	2.755	0.008
	HL on CT_max_	40	0.081	0.023	3.44	0.001
	HL and HR on CT_max_	40	9.011	2.069	4.354	0.001
	RH on CT_max_	40	1.345	0.424	3.1	0.003
	ST on CT_max_	101	0.041	0.048	0.85	0.396
	HR on CT_max_	101	1.655	0.212	7.807	0

Regarding CT_max_, models 3 and 4 exhibited a similar fit (difference in AIC value < 1). Both models indicated the effects of the three independent terms with no interaction (Fig. 2). Model 4 suggested a lower effect for RH, with respect to model 3 (RH: *N* 40, *B* 1.345, SD 0.424, *t* 3.1, *p* 0.003; HL: *N* 40, *B* 0.081, SD: 0.023, *t* 3.44, *p* 0.001), and HR (*N* 40, *B* 9.011, SD 2.069, *t* 4.354, *p* 0.001). Full results can be found in the supplementary material (Table S2 B).

### Effects of start temperature (ST) and heating rate (HR) on VT_max_ and CT_max_

**Fig. 3:**
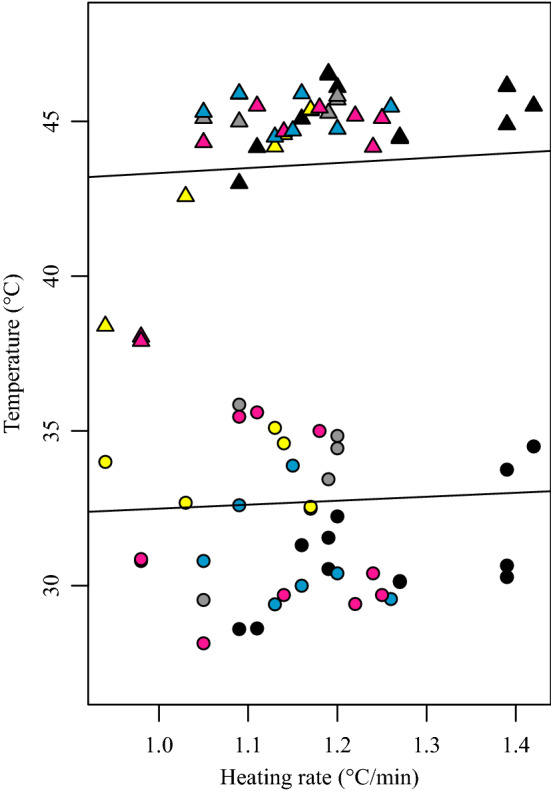
Relationships between VT_max_, CT_max_ and heating rate. Heating rates increased both the VT_max_ and CT_max_ linearly. Each point in the figure represents one ant. Circle points represent VT_max_ measures and triangle points represent CT_max_ measures (color figure online)

The models compared were: (1), intercept, (2) HR, (3) HR plus ST and (4) HR plus ST, interacting. Among the three models with similarly low AIC (Table 1), the one with lowest AIC indicated that ST had no effects on either VT_max_ (*N* 102, *B* 0.134, SD 2.467, *t* 1.47, *p* 0.143) or CT_max_ (*N* 102, *B* 0.041, SD 0.048, *t* 0.85, *p* 0.396) (Fig. S1). On the contrary, raising the HR increased both ants’ VT_max_ (*N* 102, *B* 1.313, SD 0.373, *t* 3.516, *p* 0.001) and CT_max_ (*N* 102, *B* 1.655, SD 0.212, *t* 7.807, *p* 0.000), making VT_max_ range from 30 °C to 37.8 °C and CT_max_ from 40.6 to 48 °C across the range of heating rates (Fig. 3, Table S3). The model including an interaction found no effects at all, but we stay with the effect of heating rate, since it was observable in all experiments.

## Discussion

Our measurement system allowed us to observe how physiological and behavioral thermal tolerance may combine in response to different factors. We discovered that the VT_max_ and CT_max_ of ants may describe different responses (linear vs non-linear) to changes in body size. No previous data relating VT_max_ and size from other ants or arthropod species are available to compare with ours. Lizards have shown a negative relationship of VT_max_ with body size (Camacho et al. 2018), possibly related to age. The body size of active leaf-cutting ants may relate less to age and mostly to their physical caste, where ants of different sizes dedicate more time to specific tasks. Small workers (i.e., head width up to 1.4 mm) often perform tasks within the nest (e.g., hyphae and larvae care, gardening, nest defense, Hölldobler and Wilson 1990; Wilson 1980) and related to water transportation (Ribeiro and Navas 2008). In turn, average-sized workers (head width about 2.2 mm) are most often involved in tasks that require more time outside the nest (temporal castes, Wilson 1980). That is, they explore, forage and recruit more often (Wilson 1980). Since workers of such sizes (2–2.6 mm head width) exhibited a higher VT_max_, we propose the hypothesis that within morphological castes, determined by body size, temporal castes might be formed due to their predisposition to accept higher temperatures. This seems to come at the expense of exposing themselves to higher thermal risk, since their CT_max_ did not increase in parallel. Future experiments, designed within a context of division of labor, may test the two hypotheses by comparing the VT_max_ and thermal tolerance (e.g., CT_max_ or survival time at VT_max_) of ants specifically selected when performing different tasks. Our nests were originated from wild queens and kept at room temperature. Therefore, our studied ants were never exposed to sunrays or particularly hot ant’s trails, as it would happen in the wild. In this sense, it seems unlikely that the observed increases in VT_max_ arise from adaptation or acclimation due to specific exposure to heat in captivity. Therefore, it remains to be determined how some average-sized *Atta* ants come to be more “thermally daring”.

Among hymenopterans, physiological differences are often found within morphologically defined castes (e.g., ability to follow pheromones, Robinson 2009). The separation of reproductive and working castes may be achieved by feeding the animals with different substances (Dussutour and Simpson 2009; Markin 1970; Petralia and Vinson 1978). However, no inter-castes or intra-castes differences have been reported in behavioral thermal tolerance for any hymenopteran. Yet, another example of thermally daring “special forces” might be the self-heating warrior bees of the species *Apis cerana* and *Apis mellifera, which are able to* kill Asian wasps *Vespa velutina* (Ken et al. 2005). Having thermally daring workers that maintain normal behavior at higher temperatures might benefit nests by extending foraging times during hotter periods or larger foraging areas, while the rest of the colony occupies cooler spaces (Cerdá and Retana 2000). More thermotolerant species have been found to be more abundant among Mediterranean species (Cerdá et al. 2002), and in at least one species, larger and more thermotolerant individuals forage at hotter hours of the day (Cerdá et al. 1998a, b). For leaf-cutting ants, as a consequence of spending longer times foraging at hotter temperatures, the lifespan of average-sized workers might be shortened (Mirhosseini et al. 2017; Rezende et al. 2014), compared to other workers specialized in bringing water (the smallest in the colony, Ribeiro and Navas 2008) or defending the colony (the largest ones, Powell and Clark 2004).

Our results on the CT_max_ raise considerations for the design of studies of CT_max_-size relationships, for leaf-cutting ants and other species. The homogeneous warming system used in this study (see Fig. S2 in the supplementary material for further details) prevented ants from creating large thermal gradients by raising over their legs to avoid the heating hotplate, where the temperature is often measured in studies on ants’ thermal tolerance. The existence of these gradients in laboratory assays may explain why leaf-cutting ants’ CT_max_ correlates with body size in studies using a hot plate (e.g., Ribeiro et al. 2012, Whitford and Ethershank 1975), while not in ours. Longer legs might well protect larger ants by distancing them from heating surfaces (Cerdá and Retana 2000; Sommer and Wehner 2012), but our results suggest that size does not pose further protection against rapid homogenous heating, at least within leaf-cutting ants. Yet, interspecific effects of body size on ants’ CT_max_ have been found when using a setup more similar to ours (homogenously and slowly heated vials, Baudier et al. 2015; Baudier and O’Donnell 2020). To better understand the implications of body size on the CT_max_ of ants and other arthropods, future studies might combine slow and dynamic methods for calculating the CT_max_ (Lutterschmidt and Hutchison 1997). In this way, they could evaluate how behavior (VT_max_), critical limits (CT_max_) and morphology (size) interact with the time spent at stressful/sublethal temperatures (see Castañeda et al. 2015; Rezende et al. 2020).

Hot plates are widely used for estimating the CT_max_ of arthropods but this technique might overestimate this trait. This methodological problem is difficult to evaluate when using large global databases of thermal tolerance data, such as the GlobTherm (Bennett et al. 2018), resulting in a potential underestimation of their vulnerability to high temperatures. For instance, *Pogonomyrmex desertorum* is considered one of the most thermophilic species known, with critical limits (CT_max_) up to 53 °C (Marsh 1985). In addition, other species of desert ant, heated from below (a heating mantle with a variable transformer), presented CT_max_  > 50 °C (Whitford and Ettershank 1975). Using a similar hot plate procedure, Ribeiro et al. (2012) measured CT_max_ up to 53 °C in *Atta sexdens rubropilosa*, which is not a thermophilic species, despite presenting CT_max_ similar to *P. desertorum*. Meanwhile, the maximum CT_max_ of *A. sexdens rubropilosa* in this study was 48 °C. These cases exemplify the importance of considering the heating method during the determination of thermal tolerance in arthropods.

Hydration increased both the VT_max_ and CT_max_ of leaf-cutting ants. Both traits can also increase with HL across different ectothermic vertebrates (e.g., Anurans, Anderson and Andrade 2017; Guevara-Molina et al. 2020, and lizards, Camacho et al. 2018). Instead, the humidity had only observable effects on the VT_max_ of ants. The humidity did increase the CT_max_ of termites (Woon et al. 2018), suggesting that the physiological thermal tolerance of leaf-cutting ants is less susceptible to large changes in environmental humidity. We know of no previous studies comparing the strength of body condition (hydration level) and external (relative humidity) cues on voluntary maximum temperatures, but our results suggest ants may integrate both in their behavior. These responses agree with reports of water-stressed ants selecting leaves with higher water content (Bowers and Porter 1981). However, RH did not affect the CT_max_ during heating, and ants’ VT_max_ changed most in the humid treatment. Thus, we hypothesize that this response was not anticipating higher thermal risks due to lower CT_max_, or risks derived from long-term exposures to high temperatures in a drier environment. Instead, they might simply be reacting to a better heat transmission within a more humid environment.

Ants’ VT_max_ and CT_max_ were always strongly dependent on heating rate but not start temperature. Heating rates increase the VT_max_ of bullfrogs (Guevara-Molina et al. 2020) and some lizard species (e.g., skinks), but not of other lizards (Phrynosomatids, Camacho et al. 2018). In these cases, start temperatures only marginally affected the VT_max_ of lizards, and did not affect the VT_max_ of Anurans. These observations suggest that the total heating time does not affect their behavioral responses. Yet, all of them increased their VT_max_ with heating rates, indicating that ants and other organisms might react to the time spent at stressful temperatures only. In the future, this could be evaluated using start temperatures closer to the VT_max_, and very different heating rates.

Regarding physiological thermal tolerance, heating rate may increase anurans’ CT_max_ while being detrimental for flies (Nyamukondiwa and Terblanche 2010). While heating rates have been often kept constant when measuring the CT_max_ (Lutterschmidt and Hutchison 1997), varying them still allows detecting the effects of other variables and bringing the experiments closer to the natural conditions. In nature, heating rates should vary importantly according to where individuals are (e.g., think of a sun-hit surface compared to a nest underground), and species’ ecology (e.g., terrestrial versus aquatic organisms) and physiology (e.g., more or less thermotolerant, or prone to lose water). Thus, further observations of CT_max_ are needed to find general patterns.

Concluding, the VT_max_ and CT_max_ of leaf-cutting ants may respond differently to some factors (changes in body size, humidity) and in parallel to others (start temperatures, heating rates, and hydration). Our results help understanding how behavior and thermal tolerance integrate in different situations, and also show how CT_max_ estimates may be affected by experimental design. We propose that leaf-cutting ants may have more “thermally daring” workers of average size. To continue understanding these integrative responses of organisms to temperature rises, further studies could compare the effects of these or other factors, combining dynamic and static heating systems to better understand how behavioral and physiological limits face off the temporal dimension of thermal tolerance. With few adjustments, our system could aid the observation of these limits in other small arthropods (e.g., bees, caterpillars, spiders, etc.).

## Supplementary Information

Below is the link to the electronic supplementary material.

Supplementary file1 (XLSX 113 KB)

## Data Availability

The raw data and supporting figures can be accessed here: https://doi.org/10.6084/m9.figshare.14414243

## Code availability

The raw data and supporting figures can be accessed here: https://doi.org/10.6084/m9.figshare.14414243
